# Common pathological mechanisms and therapeutic strategies in primary Sjogren’s syndrome and primary biliary cholangitis: from tissue immune microenvironment to targeted therapy

**DOI:** 10.3389/fimmu.2025.1620073

**Published:** 2025-09-29

**Authors:** Keying Ye, Xinyi Yao, Dingqi Lu, Chenfei Tan, Xinchang Wang

**Affiliations:** ^1^ The Second Clinical Medical College, Zhejiang Chinese Medical University, Hangzhou, Zhejiang, China; ^2^ Department of Gastroenterology, Sir Run Run Shaw Hospital of Zhejiang University School of Medicine, Hangzhou, Zhejiang, China; ^3^ Department of Rheumatology, The Second Affiliated Hospital of Zhejiang Chinese Medical University, Hangzhou, Zhejiang, China

**Keywords:** primary Sjogren’s syndrome, primary biliary cholangitis, serology, histology, therapy

## Abstract

Autoimmune diseases are characterized by their involvement of multiple organ systems and the presence of overlapping clinical manifestations among distinct disease entities. Both primary Sjogren’s syndrome (pSS) and primary biliary cholangitis (PBC) are chronic inflammatory disorders driven by immune-mediated injury to glandular or ductal epithelial cells. Due to shared genetic susceptibility, epidemiological patterns, and pathophysiological mechanisms, these two diseases frequently coexist in clinical practices and demonstrate a common tissue immune microenvironment. Recent advances have greatly enhanced the understanding of pSS and PBC pathogenesis, particularly regarding autoantibody profiles and pro-inflammatory cytokine expression in serum, as well as the functional activity of immune cells within affected tissues. These insights provide new perspectives and potential avenues for the development of targeted therapeutic strategies. This review examines the associations between pSS and PBC, explores the shared immunological pathways involved in disease onset and progression, and summarizes common therapeutic targets within the context of clinical treatment. The goal is to provide a comprehensive perspective that may guide future research and inform improved diagnostic and therapeutic strategies for both conditions.

## 1 Introduction

pSS and PBC are two prevalent autoimmune diseases characterized by immune-mediated damage to epithelial cells in exocrine glands or bile ducts, both classified as chronic immune-mediated epithelitis (1, 2). pSS is defined by focal lymphocytic infiltration of exocrine glands leading to structural and functional impairment of the affected tissues, and is frequently accompanied by multisystem and multiorgan involvement (1). PBC is a disease in which lymphocytic infiltration triggers inflammatory destruction of the intrahepatic portal tracts and small bile ducts, resulting in progressive hepatic fibrosis and intrahepatic cholestasis (3). Both pSS and PBC share key immunopathological features, particularly immune cell infiltration within glands or ducts, and exhibit similar pathogenic mechanisms as well as overlapping clinical manifestations.

Clinically, pSS and PBC frequently coexist within the same individual, and epidemiological studies have consistently demonstrated a relatively high comorbidity rate between these two diseases. It has been reported that approximately 19%-31% of PBC patients may be comorbid with pSS (4). Furthermore, xerostomia is observed in 43.1% of PBC patients, and histopathological examination of minor salivary glands (MSGs) in the early stages of PBC often reveals lesions that closely resemble those seen in pSS, subsequently leading to dysfunction of both lacrimal and MSGs (5, 6). In pSS, the liver represents one of the earliest extra-glandular organs to be affected. Even in pSS patients without a prior history of liver disease, clinical manifestations such as hepatomegaly and abnormal liver function tests are frequently observed, and liver biopsies in most pSS patients diagnosed with PBC reveal histological features characteristic of early-stage (Stage I) PBC (6, 7). In the absence of antimitochondrial antibody (AMA) detection and liver histological evaluation, PBC may be underdiagnosed in pSS patients, particularly in those who lack overt clinical signs of cholestasis. Therefore, the true prevalence of PBC among patients with pSS may be significantly underestimated in clinical settings.

Over the past several decades, the frequent comorbidity of pSS and PBC has garnered considerable clinical attention; however, the underlying mechanisms driving this association remain poorly understood. In recent years, a growing body of research has been dedicated to elucidating the immunopathological connections between the two diseases. This review focuses on the similarity of pSS and PBC, explores their shared pathogenic mechanisms, and systematically summarizes the overlapping therapeutic strategies observed in clinical practice. These insights may offer novel conceptual approaches for developing therapeutic strategies in pSS and PBC.

## 2 Risk factor similarities

Genetic factors play a critical role in the pathogenesis of both pSS and PBC. A Mendelian randomization analysis of the genetic data from patients with pSS and PBC suggests that these two diseases may share common genetic susceptibility loci. The onset of pSS has been associated with an increased risk of developing PBC; moreover, patients with pSS combined with autoimmune liver disease exhibit more severe disease progression and a higher mortality rate (8). Familial aggregation of pSS and PBC has also been documented. Relatives of affected individuals show a higher incidence of these diseases and are frequently affected by other autoimmune or connective tissue disorders (9, 10). At the HLA locus, pSS and PBC patients exhibit similar genetic alterations, including both susceptibility and protective alleles associated with disease onset and progression (11, 12). Additionally, genetic variants outside the HLA region also contribute to disease susceptibility, particularly those involved in T cell activation; B cell development, activation, and migration; as well as in the expression of inflammation-related signaling molecules (13, 14). These genetic variations are summarized in **Table 1** and represent key factors underlying the shared predisposition to pSS and PBC.

**Table 1:** Similar HLA genetic locus alterations.

Disease	Susceptible genes	Protective genes	Susceptibility loci outside HLA	Reference
pSS	DQA1*05:01, DQB1*02:01, DRB1*03:01	DQA1*02:01, DQA1*03:01, DQB1*05:01	IRF5, STAT4, IL12A, BLK, CXCR5, TNIP1	(12, 13, 15, 16)
PBC	DQA1*04:01, DQB1*04:02, DRB1*08:01	DQB1*03:01, DRB1*11 and 13	IL12A, IL12RB2, CSNK2B, LY6G5B, DDAH2, IRF5, SH2B3, MAPT, TYK2	(11, 17–20)

Females predominate among patients with pSS and PBC. Women’s T cells express more genes related to inflammatory and cytotoxic effector molecules compared to men’s, and several genes essential for maintaining immune tolerance are located on the X chromosome, suggesting that sex-related factors may play a critical role in disease etiology (21, 22). Liu K et al. have reported that the prevalence of pSS in women with triple X syndrome (47, XXX) is 2.9 times higher than in karyotypically normal women and 41 times higher than in males (23). Similarly, another retrospective study found that the incidence of SS in women with triple X syndrome is 2.3 times higher than in the general female population (24). Due to the unique epigenetic characteristics of the X chromosome, one X homolog undergoes inactivation in females to achieve equivalent levels of X-linked gene expression between sexes. This process, known as X-chromosome inactivation, has been proposed as a key contributor to the female predisposition to autoimmune diseases (25, 26). The frequency of X monosomy increases significantly with age in female PBC patients (27); X chromosome loss occurs more preferentially and frequently in peripheral blood mononuclear cells (PBMCs) of female PBC patients (28). A chromosome-wide association study further identified seven genes (TIMM17B, PQBP1, PIM2, SLC35A2, OTUD5, KCND1, and GRIPAP1) as potentially associated with PBC pathogenesis (29). Although X chromosome inactivation has not been extensively studied in SS, current evidence underscores the profound influence of the X chromosome on the development of both pSS and PBC.

In addition to X chromosome abnormalities and related gene expression, sex hormone levels are closely associated with the development of pSS and PBC. Both pSS and PBC predominantly affect perimenopausal and postmenopausal women, who typically exhibit reduced levels of estrogen and other hormones involved in its biosynthesis (30, 31). Since estrogen receptors are widely expressed on the surface of various immune cells, estrogen can modulate immune function by suppressing the production of pro-inflammatory cytokines from T helper type 1 (Th1) cells, stimulating anti-inflammatory cytokine secretion from Th2 cells, and regulating the balance between Th17 cells and regulatory T cells (Tregs). Consequently, dysregulated or diminished estrogen levels in perimenopausal women can enhance Th17 cell activation and impair the immunosuppressive function of Tregs, ultimately increasing susceptibility to pSS and PBC. Experimental studies using an ovariectomized murine model have demonstrated accelerated pSS progression and aggravated tissue damage; estrogen treatment significantly ameliorated inflammatory lesions in the submandibular glands (SG) of SS mice and reduced glandular lymphocyte infiltration (32). Similarly, female PBC mice displayed more severe upregulation of interferon (IFN) signaling and cholestasis compared to males (33). Moreover, the estrogen precursor dehydroepiandrosterone and its metabolites significantly reduce interleukin-8 (IL-8) and tumor necrosis factor-α (TNF-α) production by cholangiocytes and hepatocytes in PBC patients, thereby modulating the biliary immune microenvironment and alleviating hepatic inflammation (31).

Environmental exposures and infections also increase susceptibility to pSS and PBC. Lifestyle and environmental factors, including cigarette smoking, nail polish, and hair dyes, significantly elevate the risk of developing these conditions (34, 35). Viral infections, particularly hepatitis C virus (HCV) and Epstein-Barr virus (EBV), are widely recognized as potential triggers of disease onset. HCV infection not only elevates the incidence of immune cryoglobulinemia in pSS patients but also plays a major role in hepatic involvement and serves as an important pathogenic contributor in PBC (35, 36). In addition, EBV antibodies or viral genetic material, along with autoantigens homologous to EBV antigens, have been detected in salivary gland epithelial cells (SGECs) and liver tissues, as well as in PBMCs of pSS and PBC patients (37, 38). Through molecular mimicry, EBV can induce abnormal autoantigen presentation and initiate autoimmune responses, thereby establishing a strong association with the pathogenesis of both diseases. Furthermore, EBV infection enhances autoantibody production in pSS and increases the risk of lymphoma among affected individuals (39).

## 3 Serological characteristics similarities

pSS and PBC are characterized by distinct serological biomarkers. pSS is typically diagnosed by the presence of serum anti-SSA and/or anti-SSB antibodies, together with an antinuclear antibody (ANA) titer of ≥1:320 (40). PBC is defined by the presence of serum anti-mitochondrial antibodies (AMA) at titers >1:40, typically accompanied by biochemical evidence of hepatic dysfunction, including elevated alkaline phosphatase (ALP) and γ-glutamyl transferase (GGT) levels (3, 41).

The ANA profile represents a spectrum of autoantibodies directed against various intracellular components, including nuclear, cytoplasmic, and cytoskeletal antigens. This profile encompasses ANA, AMA, anti-SSA, and anti-SSB antibodies, which serve as important biomarkers for the diagnosis of various autoimmune diseases and for the monitoring of disease activity.

In pSS, autoantibodies targeting SGECs, particularly anti-SSA (Ro52 and Ro60) and anti-SSB antibodies, are considered key mediators of autoimmunity. Anti-SSB antibodies generally coexist with anti-SSA antibodies, whereas the elevated titers of anti-SSA antibodies are closely associated with immune activation and reflect both the degree of inflammation and disease activity in pSS (42). Both in peripheral blood and MSGs, the detection rate of anti-SSA antibodies is significantly higher in pSS patients compared with non-pSS individuals, underscoring their diagnostic specificity (43). Anti-Ro52 antibodies are strongly correlated with increased serum levels of IgA and IgG (44). Patients with isolated anti-Ro52 positivity often present with higher rheumatoid factor (RF), while those positive for both anti-Ro52 and anti-Ro60 demonstrate more pronounced glandular inflammation, excessive B-cell activation, and elevated related biomarkers (45). Furthermore, the expression of interferon-stimulated genes is positively correlated with the presence of anti-Ro60 antibody (46).

AMA, as a specific biomarker for PBC patients, is capable of recognizing PDC-E2 antigen on the surface of biliary epithelial cells (BECs). Since PDC-E2 is also present on SGECs, AMA can similarly bind to these cells as well and mediate immune-mediated damage to SGECs. In addition to AMA, the 210 kDa glycoprotein (gp210), a nuclear pore membrane component, and the 100 kDa glycoprotein (sp100), associated with nuclear dots, are recognized as PBC-specific nuclear antibodies. AMA is detectable in the vast majority of PBC patients, and AMA-positive individuals typically exhibit elevated serum IgM levels. Anti-gp210 and anti-sp100 antibodies are detected in approximately 34% and 26% of patients, respectively. Although these markers demonstrate high diagnostic specificity, their sensitivity is relatively limited. Notably, gp210 antibody positivity correlates with significantly higher serum levels of ALT, GGT, ALP, and AST, as well as with poorer clinical prognosis. Thus, detection of anti-gp210 and anti-sp100 antibodies is of considerable clinical significance in PBC (47). Incorporating these tests into clinical practice can improve diagnostic accuracy in AMA-negative PBC, reduce the reliance on invasive liver biopsy, and enhance the overall reliability of non-invasive diagnostic approaches (48). Nevertheless, additional candidate biomarkers for the diagnosis of AMA-negative PBC remain under active investigation.

Anti-centromere antibodies (ACA) are detected in both pSS and PBC and may serve as an important serological marker for overlap syndromes, such as pSS combined with PBC onset (49, 50). In a cohort study involving 241 individuals with SS, systemic sclerosis, or PBC, as well as healthy controls, ACA was detected in 15% of SS patients and 20% of PBC patients (51). Among patients with pSS, ACA positivity is associated with markedly increased salivary gland fibrosis and more severe secretory gland dysfunction (52, 53), and distinct serological profiles with higher disease activity compared to ACA-negative individuals (54). In PBC, anti-gp210 antibody positivity correlates with the severity of interface hepatitis and lobular inflammation, while ACA-positive patients tend to follow a portal hypertension–dominant disease course accompanied by pronounced ductular reactions (55). Fibrotic changes predominate in ACA antibody-positive pSS, and ACA-positive PBC can predict a poor prognosis in the diagnosis of PBC (53, 56). Thus, the detection of ACA in combination with histological findings provides valuable diagnostic and prognostic information in both pSS and PBC.

## 4 Histologic similarities

Both pSS and PBC are characterized by varying degrees of progressive target-organ damage during disease progression and exhibit notable similarities in their histopathological features.

In pSS, histopathological changes in the minor salivary glands (MSGs) are characterized by lymphocytic infiltration surrounding the salivary ducts, leading to acinar atrophy and progressive destruction of glandular tissue. The infiltrating cells are predominantly CD4^+^ T lymphocytes, accompanied by B lymphocytes and plasma cells. Focal lymphocytic sialadenitis (FLS) is a defining pathological hallmark of pSS, and the presence of ≥50 lymphocytes within a 4 mm² area of salivary gland tissue constitutes one focus (FS = 1). A focus score (FS) ≥1 per 4 mm², in combination with serological findings, is considered diagnostic for pSS (40). As the disease progresses, extensive lymphocyte accumulation around ducts and invasion into follicles or epithelial cells result in follicular atrophy and disappearance, and ductal epithelial hyperplasia or luminal dilation. In advanced stages, ductal stenosis, obstruction, and fibrosis may occur, ultimately resulting in complete loss of exocrine function (57, 58). Notably, 10–30% of pSS patients develop ectopic germinal centers (eGCs) in their MSGs, which substantially increases the risk of lymphoma (59).

Patients with PBC progress through distinct pathological stages, including peribiliary inflammation, hepatic fibrosis, and ultimately cirrhosis. In the early phase, the liver demonstrates inflammatory changes resembling those seen in pSS, characterized by dense lymphocytic and plasma cell infiltration surrounding the bile ducts (60). This inflammatory infiltrate induces edema and degeneration of biliary epithelial cells, resulting in severe epithelial injury, disruption of the ductal basement membrane, lymphoid aggregates, and ductopenia, accompanied by mild to moderate hepatocellular necrosis, cellular swelling, and cholestasis. As the disease advances, inflammatory granulomas develop and contribute to the gradual loss of small intrahepatic bile ducts (2, 61). In stages III and IV, confluent fibrosis extends throughout the portal tracts and adjacent tissues, accompanied by progressive periportal cholestasis. Obstruction of the canalicular bile ducts ultimately results in cirrhosis and the formation of regenerative nodules. Moreover, owing to persistent lymphocytic aggregation, tertiary lymphoid structures (TLS), resembling ectopic germinal centers, emerge within the hepatic tissue of PBC patients (61, 62).

## 5 Tissue immune microenvironment similarities

**Figure 1:**
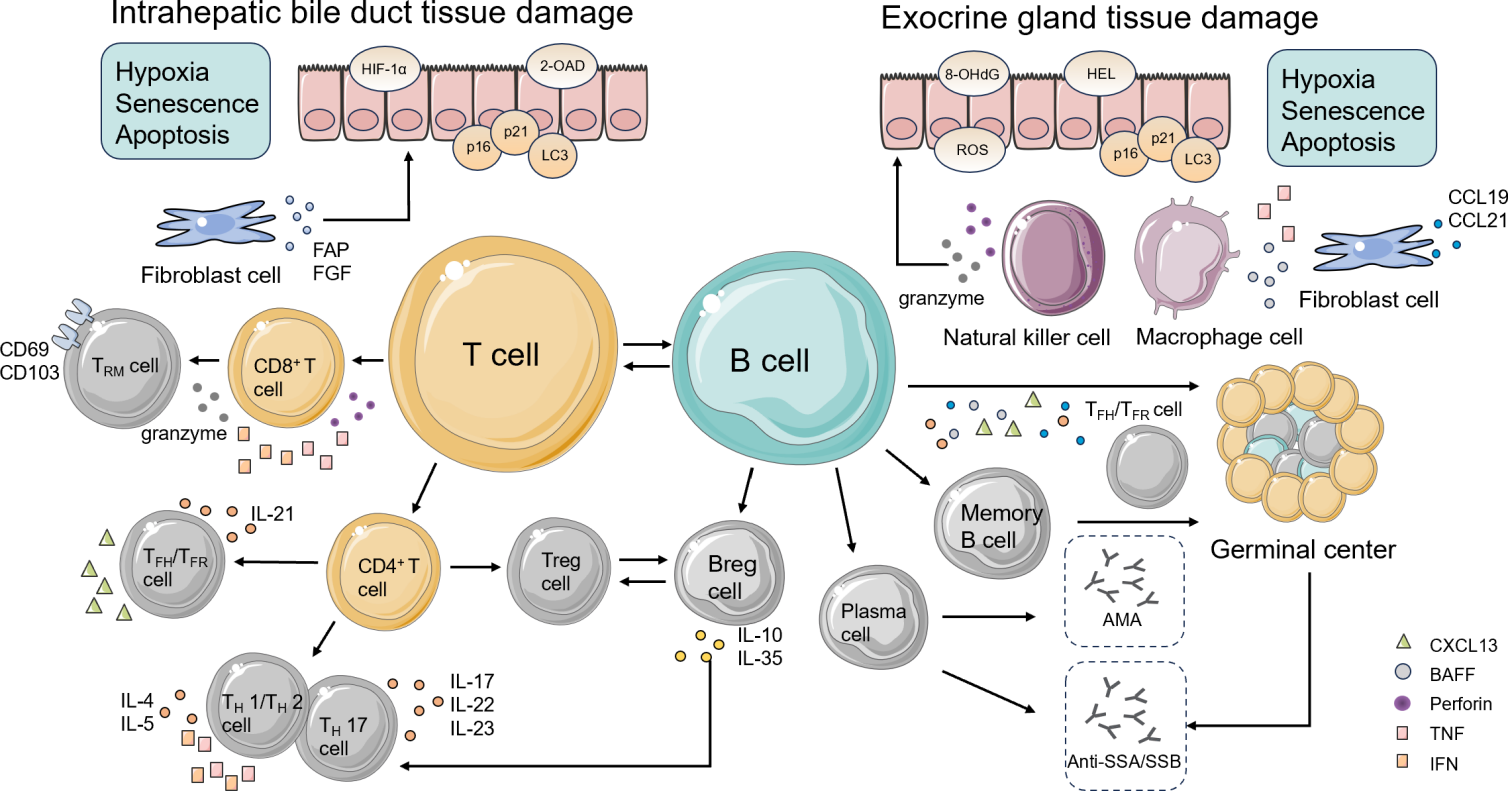
The exocrine gland tissue in pSS and the bile duct tissue in PBC exhibit similar immune microenvironments. In this context, epithelial cells display aberrant expression of genes associated with hypoxia, senescence, and autophagy. Both SGECs and BECs abnormally present self-antigens, thereby becoming targets of autoimmune attack and creating a parallel immunopathological landscape. Fibroblasts release pro-inflammatory mediators and chemokines that recruit inflammatory cells; in PBC, aberrant expression of FAP and FGF further accelerates tissue fibrosis. NK cells show enhanced cytotoxicity, secreting perforin and granzyme, and infiltrate both MSGs and hepatic tissues, where they drive epithelial injury. Widespread macrophage infiltration upregulates genes linked to inflammation, tissue injury, and metabolic dysfunction, amplifying lymphocyte-mediated immune responses through co-stimulatory mechanisms. T and B lymphocytes both contribute to the initiation and persistence of inflammatory infiltrates in MSGs and bile ducts. T cells differentiate into distinct subsets and secrete cytokines, including IFNs, ILs, and TNFs, that perpetuate tissue inflammation and injury. The regulatory capacity of Tregs to maintain immune balance and homeostasis is impaired, while an imbalance in the Tfh/Tfr ratio leads to aberrant B cell activation. Enhanced cytotoxic activity of CD8^+^ T cells not only induces epithelial damage but also promotes B-cell antibody production. Following differentiation into Trm cells, B cells persist within tissues, sustaining local immune activation. Ultimately, B cells and plasma cells in eGCs generate diverse autoantibodies that exacerbate both local and systemic tissue damage. pSS, primary Sjogren’s syndrome; PBC, primary biliary cholangitis; SGECs, salivary gland epithelial cells; BECs, biliary epithelial cells; FAP, fibroblast activation protein; FGF, fibroblast growth factor; NK cells, natural killer cells; MSGs, minor salivary glands; IFN, interferon; ILs, interleukins; TNF, tumor necrosis factor; BAFF, B cell activating factor; Th, T helper; Tregs, regulatory T cells; Tfh, T follicular helper; Tfr, follicular regulatory T cells; Trm, tissue-resident memory T cells; HIF-1α, hypoxia-inducible factor 1α; 2-OAD, 2-oxoglutarate dehydrogenase; 8-OHdG, 8-hydroxy-2’-deoxyguanosine; HEL, hexanoyl lysine; ROS, reactive oxygen species; CCL, chemokine (C-C motif) ligand; CXCL, chemokine C-X-C motif ligand; CD, cluster of differentiation; AMA, antimitochondrial antibodies; anti-SSA/SSB, anti-Sjogren’s syndrome A/B; eGCs, ectopic germinal centers.

The similarity in target tissue damage observed in pSS and PBC is primarily attributable to shared dysregulation of the immune microenvironment. This dysregulation is characterized by impaired function of epithelial cells and fibroblasts, aberrant activation of T and B lymphocytes, and dysregulated expression of various inflammatory mediators and cytokines (**Figure 1**).

### 5.1 Non-immune tissue cells

#### 5.1.1 Epithelial cell

SGECs and BECs play a pivotal role in shaping the immune-dysregulated microenvironment. In pSS and PBC tissues, epithelial cells facilitate autoantibody recognition through their antigen-presenting function. They also produce pro-inflammatory cytokines, adhesion molecules, and chemokines, which promote B-cell activation and T-cell infiltration. These signals recruit and retain immune cells in the tissue, ultimately sustaining chronic inflammation.

Within epithelial cells, increased production of reactive oxygen species (ROS) and exacerbated mitochondrial damage disrupt microcirculation and mitochondrial metabolism, leading to heightened local tissue hypoxia and promoting cellular apoptosis. In the MSGs of pSS patients, oxidative stress markers such as 8-hydroxy-2’-deoxyguanosine (8-OHdG) and hexanoyl-lysine (HEL) are elevated, reflecting a pro-oxidant state in the tissue (63). In the liver tissue of PBC patients, 2-oxoacid dehydrogenase (2-OAD) activity is impaired, hypoxia-inducible factor 1α (HIF-1α) secretion is increased, and PDC-E2 fails to properly participate in intracellular redox reactions. These alterations exacerbate mitochondrial dysfunction and aggravate hepatic hypoxia (64). The resulting hypoxic microenvironment promotes the release of inflammatory factors from immune cells, causing abnormal macrophage activation, Th17 differentiation, and an imbalance in Treg cell populations. Collectively, these changes enhance lymphocyte infiltration and tissue injury, thereby accelerating hepatic fibrosis and worsening cholestasis in PBC patients.

Dysregulation of cellular senescence and autophagy mechanisms is closely implicated in the pathogenesis of autoimmune diseases. Cellular senescence is a manifestation of cessation of cell proliferation processes and substantial alterations in gene expression, while autophagy is a protective catabolic process that degrades misfolded proteins and damaged organelles. Both processes serve to delay apoptosis by mitigating cellular damage, stabilizing cellular homeostasis, and maintaining metabolic homeostasis. In pSS patients, however, the expression of senescence markers p16 and p21 is significantly elevated in MSGs, and epithelial cells exhibit impaired self-renewal capacity. Even after the resolution of inflammatory infiltration in the MSGs, salivary gland function fails to recover (65). Autophagy-related genes are upregulated in the MSGs of pSS patients, and the increased expression of LC3, an autophagosome marker in lymphocytes, promotes the secretion of IL-23 and IL-21, thereby exacerbating inflammation and tissue damage (66). Animal experiments have shown that correcting autophagy dysregulation in aged mice reduces the formation of TLS in SG and ameliorates glandular pathology (67). In PBC patients, BECs in damaged small bile ducts display significantly shortened telomeres and markedly elevated expression of the senescence-associated secretory phenotype (68). LC3 is also strongly expressed in BECs and co-localizes with senescence markers p16 and p21 in inflamed bile ducts (69). Moreover, p16 expression is closely correlated with the staging of PBC (70).

In pSS and PBC patients, interconnected mechanisms, including cellular senescence, dysregulated autophagy, and oxidative stress, are interconnected, leading to widespread epithelial cell damage and enhanced immune responses. These factors collectively accelerate disease progression and identify potential targets for therapeutic intervention.

#### 5.1.2 Fibroblast

Fibroblasts are widely distributed across tissues and secrete cytokines, including B lymphocyte stimulator (BLyS) and IL-7, chemokines like CXCL13, CCL19, and CCL21, and growth factors. These factors collectively facilitate lymphocyte transendothelial migration, promote cell development, proliferation, and differentiation, and help maintain tissue structural integrity. In the context of autoimmune diseases, inflammation-associated fibroblasts exhibit dysregulated cytokine production and altered functional responses through the activation of downstream signaling pathways.

In pSS patients, fibroblasts in the MSGs form an intricate network of interactions with surrounding cells. These fibroblasts exhibit significant downregulation of signaling pathways related to SGECs’ growth and extracellular matrix components, such as collagen and laminin, while pro-inflammatory pathways are markedly upregulated. This dysregulation impairs epithelial regeneration and glandular development, triggering a cascade of inflammatory responses in pSS (71). In pSS, fibroblasts aberrantly express chemokines such as CCL21 and CCL19, recruiting lymphocytes and contributing to the formation of TLS under the influence of IL-22 and lymphotoxins, thereby exacerbating inflammatory infiltration within pSS tissues (72, 73). Additionally, fibroblasts express CD21, a complement receptor 2, or the EBV virus receptor. EBV can exploit this receptor to abnormally activate B cells, which may play a role in triggering pSS in otherwise healthy individuals following EBV infection (74).

In PBC patients, fibroblast-like cells and stromal deposits accumulate in the periductal regions of proliferating bile ducts, representing a key contributor to hepatic fibrogenesis. The inflammatory environment within the liver upregulates the expression of fibroblast activation protein on fibroblasts (75). In concert with pro-fibrotic activation of hepatic stellate cells and extracellular matrix deposition, this process exacerbates both fibrotic histopathology and inflammatory infiltration, establishing a positive feedback loop that perpetuates tissue scarring and inflammation. In addition, fibroblast growth factor 19 interacts with the bile acid receptor FXR to downregulate hepatic bile acid levels. However, in PBC, the abnormal expression of fibroblast growth factor 19 and dysregulated FXR interactions disrupt bile acid homeostasis, intensifying cholestasis and promoting liver injury (76).

### 5.2 Immunocyte

#### 5.2.1 Natural killer cells

Natural killer (NK) cells are innate immune lymphocytes capable of exerting cytotoxic effects and releasing cytokines without prior antigen exposure. Their cytotoxic activity promotes apoptosis of SGECs and BECs through the secretion of perforin and granzyme, as well as through activation of the Fas/FasL signaling pathway.

Both pSS and PBC demonstrate significant genetic enrichment related to NK cells (4). In the MSGs of pSS patients and the liver of PBC patients, NK cells accumulate around infiltrating lesions, exhibit heightened cytotoxicity, and show markedly increased perforin expression (77, 78). Guided by the CX3CL1/CX3CR1 axis, NK cells migrate to and persist within MSGs and hepatic tissues; they further promote local immune infiltration through CCL3 and CCL5, thereby reinforcing the abnormal immune microenvironment, amplifying local immune responses, and accelerating disease progression in both pSS and PBC (79).

#### 5.2.2 Monocyte-macrophage cell

Macrophages regulate the immune responses by secreting inflammatory cytokines and chemokines that coordinate interactions among various components of the immune system. Through Toll-like receptor (TLR) signaling, they provide co-stimulatory signals that recruit lymphocytes and amplify immune activation. Critically, macrophages are indispensable for the activation of CD4^+^ T cells and for supporting the development and maturation of B cells.

In pSS patients, the proportion of macrophages is elevated in damaged glandular tissues, where they persist around autoimmune lesions (80). Infiltrating M1 macrophages amplify the expression of genes associated with inflammation, tissue injury, and metabolic dysregulation, while releasing pro-inflammatory cytokines such as IL-6, TNF-α, and IL-12. Although M2 macrophages secrete anti-inflammatory mediators, accumulating evidence indicates that they may emerge during later disease stages and contribute to glandular fibrosis in the context of chronic inflammation (81). Kupffer cells, the resident macrophages of the liver, also play a pivotal role in PBC. In these patients, macrophages exhibit upregulated expression of Engulfment and Cell Motility 1 (ELMO1), which activates the NF-κB signaling pathway, promotes macrophage migration into the hepatic tissue, and enhances both cytokine secretion and phagocytic activity (82). Through these mechanisms, macrophages intensify local inflammatory responses and aggravate cholestasis.

#### 5.2.3 T-lymphocyte cells

T and B lymphocytes act in concert to drive inflammatory infiltration within glandular and tissue sites. Aberrant activation of CD4^+^ T cells promotes tissue inflammation through the secretion of pro-inflammatory cytokines such as IFN-γ, IL-17, and TNF-α, and facilitates the differentiation of CD8^+^ T cells into cytotoxic effectors that mediate tissue damage. In addition, T cells are critically involved in promoting B cell development, proliferation, and activation, as well as in the formation of TLS, thereby enhancing autoantibody production and further aggravating tissue injury.

##### 5.2.3.1 Th1 and Th2 cells

Th1 cells primarily induce epithelial cell apoptosis through the secretion of pro-inflammatory cytokines such as IFN-γ and TNF-α. They also facilitate antigen clearance through enhancing antigen presentation and promoting the activation of immune cells. Th2 cells promote B cell activation and antibody production via the secretion of cytokines, including IL-4 and IL-5, thereby playing a pivotal role in antibody-mediated immune responses. However, when the activity of both Th1 and Th2 subsets is abnormally elevated, the immune system may aberrantly present self-antigens and initiate destructive responses against host tissues, ultimately contributing to the development of autoimmune diseases.

In patients with pSS and PBC, cytokines secreted by Th1 and Th2 cells selectively recruit helper and cytotoxic T cell subsets, promote aberrant Th17 differentiation, and induce abnormal activation of B cells and NK cells. These events disrupt lymphocyte distribution and tissue homeostasis, ultimately leading to increased IgM and IgA production. In the MSGs of pSS patients, infiltration of Th1 and Th2 cells is elevated, accompanied by significantly increased transcription of cytokine and chemokine genes associated with these subsets (83). Similarly, in PBC, Th1- and Th2-related transcripts are upregulated and expressed disproportionately in both blood and liver (84). In the early stage of PBC, IFN-γ- and CXCR3-expressing Th1 cells accumulate around damaged bile ducts, releasing inflammatory mediators that sustain hepatic inflammation by recruiting lymphocytes and other immune cells. As the disease progresses, portal lymphocytes differentiate into Th1 cells, becoming key mediators of persistent bile duct injury (85). Thus, the heightened expression and imbalance of Th1/Th2 subsets perpetuate chronic inflammation and exacerbate tissue damage, playing a central role in the pathogenesis and progression of both pSS and PBC.

##### 5.2.3.2 Th17 and Treg cells

Th17 cells mediate pro-inflammatory responses by secreting cytokines such as IL-17, IL-22, and IL-23, while they also recruit lymphocytes to initiate and amplify local inflammation. In patients with pSS, IL-17 levels are significantly elevated in peripheral blood, and IL-17-producing cells are abundantly infiltrated within MSG lesions (86). Experimental knockdown of ubiquitin-specific peptidase 18 in murine models reduces the proportion of Th17 subsets within salivary glands, restores Treg subsets in salivary glands, improves salivary flow rates, and attenuates lymphocytic infiltration (87). In advanced PBC, IL-17^+^ cells are markedly increased in the liver and accumulate around BECs (88). The IL-23/IL-17 axis promotes lymphocyte differentiation into Th17 cells and enhances IFN-γ production, thereby skewing immune responses toward a Th17-dominant phenotype and sustaining hepatic inflammation. BECs express IL-17 receptors that respond directly to IL-17, further promoting autoinflammation. Moreover, IL-17 derived from PBC patients strongly stimulates the proliferation of hepatic stellate cells, thereby accelerating hepatic fibrosis (89).

Tregs, which are central to maintaining immunoregulatory balance and immune homeostasis, suppress excessive immune responses by modulating the inflammatory milieu or by directly inducing apoptosis in T cells. Treg deficiency has been closely implicated in the early onset of pSS. In murine models, SATB1 gene knockout results in Treg depletion, which exacerbates lymphocytic infiltration in the SGs and induces SS-like manifestations (90). In PBC, the hepatic microenvironment is enriched with pro-inflammatory cytokines such as IL-6, IFN-γ, and IL-1β, but is deficient in IL-2. This cytokine imbalance destabilizes Tregs and compromises their suppressive capacity against lymphocyte proliferation (91). Clinically, PBC patients exhibit markedly reduced circulating Treg frequencies, which correlate with elevated serum ALT levels and increased autoantibody expression (48). Importantly, patients with overlapping pSS and PBC present a more profound Th17/Treg imbalance and more severe hepatic dysfunction (92). Taken together, Treg deficiency or functional impairment represents a critical factor driving immune dysregulation and disease progression in both pSS and PBC.

Recent studies have identified FoxP3 demethylation as a key mechanism impairing Treg function (93). In pSS patients, the number of FoxP3^+^ Tregs in MSGs correlates positively with the degree of inflammatory infiltration (94). FoxP3 demethylation exacerbates PBC progression by compromising Treg function (95). Moreover, complete ablation of FoxP3^+^ Tregs in animal models induces a PBC-like phenotype characterized by anti-AMA antibody production (96). Loss of Treg-mediated suppression promotes the expansion of pathogenic Th17 cells and excessive production of Th17 cytokines, while Treg depletion amplifies pro-inflammatory signaling across diverse immune cell populations. Conversely, restoring functional Tregs within glandular and hepatic tissues can reestablish a balanced lymphocyte microenvironment, attenuate excessive inflammation, and alleviate disease severity, thereby representing a promising therapeutic approach for both pSS and PBC.

##### 5.2.3.3 Tfh and Tfr cells

T follicular helper (Tfh) cells secrete cytokines that enhance the activation and proliferation of B cells, which in turn regulate the development of B cells towards mature plasma cells and memory cells and promote TLS formation and B cell antibody production. Conversely, follicular regulatory T (Tfr) cells, a specialized subset of Tregs, suppress immune responses by secreting immunosuppressive cytokines or competing with Tfh cells for ligand engagement, thereby limiting B cell differentiation and functional activity.

In both pSS and PBC, Tfh cells exhibit aberrant upregulation of inducible T cell costimulator (ICOS), programmed death receptor 1 (PD-1), enhancer of zeste homolog 2 (EZH2), and chemokines (97, 98). This dysregulation, mediated by enhanced STAT phosphorylation under the regulation of IL-21 and Bcl6, drives quantitative and functional abnormalities in Tfh cells while concurrently suppressing Tfr cell proliferation and activity. As a result, B cells become excessively activated within tissues, producing increased autoantibodies and amplifying autoimmune responses. In pSS, Tfh and B cells accumulate in the MSGs at later stages and display a synchronized developmental trajectory. The frequency of activated Tfh cells correlates positively with anti-SSB antibody levels and serum IgG concentrations (99), highlighting the pathogenic significance of Tfh-B cell interactions in disease progression. In PBC, an imbalanced Tfr/Tfh ratio promotes aberrant B cell activation, excessive IgM secretion, enhanced lymphocyte chemotaxis, and increased death of normal cells. Impaired Tfr function also diminishes inhibition of TLS formation, further elevating local antibody production. Restoring the Tfr/Tfh balance improves GC-related factor expression, reduces tissue lymphocyte infiltration, and may mitigate autoimmune pathology. Currently, the Tfr/Tfh ratio has been proposed as a potential biomarker to differentiate clinical manifestations of pSS and PBC; however, its specificity and sensitivity require further validation.

##### 5.2.3.4 CD8^+^ effector T cells

CD8^+^ effector T cells, also known as CD8^+^ cytotoxic T lymphocytes, can specifically recognize self-antigens presented by MHC class I molecules. By releasing cytokines such as IFN-γ and TNF, together with cytotoxic effector molecules including granzyme and perforin, they directly induce lysis of target cells. In pSS and PBC patients, tissue-infiltrating CD8^+^ T cells mediate direct cytotoxic effects on epithelial cells, thereby amplifying local inflammatory responses and aggravating epithelial injury.

In pSS patients, CD8^+^ T lymphocytes in the MSGs exhibit markedly elevated expression of granzyme K and heat shock proteins, enhancing their cytotoxicity against self-antigens and contributing to persistent stress responses and low-grade glandular inflammation (100). In early-stage PBC, CD8^+^ T cells recognize MHC class I-restricted PDC-E2 epitopes on BECs, mediating aberrant antigen presentation and inducing BEC apoptosis. Under the influence of excessive IFN-γ secretion by CD4^+^ T cells, these CD8^+^ T cells further amplify bile duct inflammation, ultimately leading to epithelial injury (101). In advanced disease, an imbalance between CD8^+^ T cells and Tregs, together with a pronounced infiltration of CXCR6^+^CD8^+^ T cells in the peribiliary region, drives aggravated hepatic inflammation, intensifies extensive bile duct injury, and accelerates hepatic fibrosis in PBC patients (102).

CD8^+^ tissue-resident memory T cells (Trm) represent a distinct memory T cell subset that resides independently of the peripheral circulation. Residing long-term in peripheral non-lymphoid tissues, they provide rapid and localized defense against reinfection by specifically recognizing PDC-E2, directly eliminating target cells, or secreting antiviral cytokines. In the SGs of SS murine models, Trm cells exhibit hyperproliferation and hyperactivation, producing high levels of inflammatory mediators, recruiting CXCR3^+^ T cells, and accelerating epithelial cell injury through the Fas/FasL pathway and cytotoxic mechanisms (103). CD8^+^ Trm cells localize within the MSG epithelium via integrin CD103-mediated adhesion, while CD69 expression enables them to resist the chemotactic gradient of sphingosine-1-phosphate in blood and lymphatic vessels, thereby preventing their recirculation (104). In the specialized hepatic immune microenvironment of PBC patients, CD8^+^ T cells upregulate E-cadherin, which interacts with the E-cadherin/β-catenin complex to form adhesive junctions. This facilitates their accumulation around BECs and promotes retention within bile ducts, thereby modulating lymphocyte infiltration and contributing to hepatic inflammation and fibrosis (105).

#### 5.2.4 B-lymphocyte cells

##### 5.2.4.1 Breg

Regulatory B cells (Bregs) constitute an immunosuppressive B cell subset that plays a critical role in maintaining immune tolerance and preventing autoimmunity. They primarily mediate their immunoregulatory effects through the secretion of anti-inflammatory cytokines, including IL-10 and IL-35, in response to immune activation (106). The key functions of Bregs encompass the inhibition of pro-inflammatory Th1 and Th17 cell differentiation, suppression of Tfh cell responses, and the potential induction of Treg expansion (107).

In autoimmune conditions such as pSS, the frequency of Breg cells shows a progressive decline. And this loss of immunosuppressive function parallels Tfh cell expansion, disruption of the inflammatory milieu, and increased disease activity (106). Some studies have reported an increased proportion of CD19^+^CD24^hi^CD38^hi^ Breg cells in pSS patients during both active and inactive phases of the disease (108), which may represent a compensatory immunoregulatory mechanism. However, whether these cells retain full functional capacity remains uncertain. In PBC, peripheral Breg cell numbers are increased, but their function is impaired, resulting in excessive activation of Th1 and Th17 cells and suppression of Treg activity. These functional abnormalities further exacerbate disease severity and correlate with elevated biochemical indicators, such as ALP (109).

##### 5.2.4.2 Memory B cells

Memory B cells, as the principal carriers of adaptive immune memory, exhibit pronounced abnormalities in both distribution and function in patients with autoimmune diseases. Upon antigenic stimulation, naïve B cells differentiate into plasma cells or memory B cells. In pSS and PBC, chemokines secreted by epithelial cells within inflamed tissues recruit circulating memory B cells to the affected glands and promote their local retention. In addition to enhancing CXCL13-mediated B cell chemotaxis, memory B cells also participate in the formation of eGCs with the support of Tfh cells, thereby further amplifying B cell activation and sustaining local autoimmune responses.

In patients with pSS, the proportion of CD27^+^ memory B cells in peripheral blood is markedly reduced; in contrast, a pronounced accumulation of memory B cells is observed within affected MSG tissues, suggesting a potential redistribution (110). This chemotactic behavior is likely mediated by the elevated expression of chemokine receptors CXCR4 and CXCR5, which respond to gradients of CXCL12 and CXCL13 secreted by epithelial cells in the inflamed glands (111). Within the exocrine glands of SS patients, memory B cells further promote the formation of GC structures, thereby amplifying B cell chemotaxis and driving lymphocytic infiltration (112). Similarly, in PBC patients, the frequency of circulating CD27^+^CD19^+^ memory B cells is significantly reduced compared with healthy controls, likely reflecting antigen-specific memory B cell redistribution from lymphoid tissues to sites of inflammation and away from the peripheral circulation (113).

##### 5.2.4.3 Plasma cells

Plasma cells (PCs) are terminally differentiated B cells that serve as the principal source of antibodies and immunoglobulins. Their abundance and functional activity are closely associated with the serological profiles and pathological damage observed in autoimmune diseases.

The survival and functionality of PCs are highly dependent on microenvironmental signals, particularly BAFF and IL-6. BAFF, induced by type I interferons, provides essential survival signals to PCs (114). PCs also exhibit chemotaxis toward sites rich in CXCL12 and IL-6, relying on these factors to sustain long-term survival and continuous antibody secretion (115). In the context of autoimmune pathology, T cells secrete IL-21 and ICOS to drive B cell differentiation into PCs, while BAFF overexpression further maintains their persistence. With assistance from Tfh cells, PCs potentiate autoimmune responses through the production of copious autoantibodies and immunoglobulins (116, 117).

Aberrant activation of PCs represents a key factor underlying the elevated titers of autoantibodies observed in patients with pSS and PBC. In patients with pSS, PCs are recruited into the MSGs via CXCL12-mediated chemotaxis and distribute across different glandular regions. The IL-6-enriched microenvironment provides a survival niche that sustains prolonged secretion of antibodies, including IgA and IgG, and contributes to increased serum ANA titers (115). In PBC, the frequency of PCs producing PDC-E2-specific antibodies is markedly improved. With additional support from Tfh cells and other cytokines, this dysregulated activation exacerbates disease activity and drives abnormalities in biochemical markers (118).

Immunophenotypic analyses have demonstrated the coexistence of IgA- and IgG-expressing PCs in the MSG tissues of patients with pSS. IgA^+^ PCs tend to form small clusters adjacent to ductal and epithelium, whereas IgG^+^ PCs are distributed throughout both the central and peripheral regions of larger focal infiltrates (115). The extent of lymphocytic infiltration influences both the clustering of PCs and the levels of IgG expression. In PBC, elevated serum IgM and IgG levels can be attributed to enhanced T–B cell interactions mediated by lymphokines. In this process, activated T cells secrete B cell growth factor and B cell differentiation factor, which promote B cell proliferation and ultimately drive their differentiation into PCs capable of secreting immunoglobulins, thereby contributing to increased immunoglobulin production (115).

Notably, the coexistence of PBC and pSS is closely linked to elevated IgM levels. A retrospective analysis of 82 patients with concomitant pSS and PBC demonstrated that the frequency of increased IgM in PBC patients with SS (53.7%) was markedly higher than in the pSS-only group (6.1%) (119). In addition, anti-SSA-positive PBC patients showed higher serum bilirubin and IgM levels, accompanied by more severe histological progression (120). Thus, elevated IgM represents a significant risk factor for the development of PBC in the context of pSS. Collectively, these observations underscore the pivotal role of abnormal PC activation and autoantibody production in the immunopathogenesis of both diseases.

##### 5.2.4.4 Ectopic germinal centers

Within the tissue immune microenvironment, inflammatory mediators drive aberrant activation of B lymphocytes and their migration into non-lymphoid tissues. In these sites, B cells interact with T cells, GC B cells, and the follicular dendritic cell network to promote the formation of eGCs. These structures, also referred to as tertiary lymphoid organs or ectopic lymphoid structures, play a pivotal role in sustaining chronic inflammation and autoimmunity.

Aberrant activation and differentiation of GC B cells constitute a central mechanism driving disease progression, regulated by a complex network of cytokine signaling and intercellular interactions. Elevated expression of CXCR4, CXCL12, and CXCL13 facilitates extensive lymphocyte infiltration into eGCs, aggravating glandular destruction and functional impairment. At the same time, the accumulation of autoantibodies facilitates immune complex formation, which can trigger diverse extra-glandular manifestations (121, 122). Plasmacytoid dendritic cells secrete IFN-I, thereby inducing the production of BAFF. BAFF not only enhances B-cell survival but also serves as a bridge between innate and adaptive immunity, sustaining chronic B-cell hyperactivation (123). Elevated BAFF levels may further sustain the activation and local accumulation of autoreactive B cells, ultimately fostering the maturation and maintenance of eGCs (124).

eGCs exhibit a secondary lymphoid organ-like structure, comprising follicular dendritic cell networks and high endothelial venules. This specialized microenvironment is conducive to high-frequency somatic hypermutation, antibody class switching, and efficient autoantibody production, and also markedly increases the risk of lymphomagenesis (124, 125).

The progression of pSS is characterized by increased periductal cellular infiltration and the establishment of eGCs within salivary glands, accompanied by autoantibody production. In MSGs, these eGCs exhibit GC-like architecture that drives markedly elevated levels of anti-SSA/SSB antibodies and RF. They are also associated with increased concentrations of local and systemic pro-inflammatory mediators, including CCL11, IFN-γ, and BAFF, thereby substantially elevating the risk of lymphomagenesis (126, 127). Similarly, in PBC, eGCs represent a hallmark feature characterized by lymphocyte accumulation in portal tracts, with their maturation correlating closely with disease-associated inflammation and fibrosis. Dysregulation of the Tfr/Tfh ratio in PBC patients impairs the inhibitory function of Tfr cells on GC formation. This loss of regulation, together with a cellular phenotype defined by high expression of CXCR5, PD-1, ICOS, and Bcl6, further exacerbates lymphocyte infiltration and epithelial cell death, exacerbating disease pathology (98).

### 5.3 The similarities in cytokines and chemical signaling molecules

#### 5.3.1 Interleukin

ILs are cytokines secreted by diverse immune cells that regulate immune responses and inflammation. They also facilitate antigen presentation and promote the proliferation and differentiation of immune cells.

In the MSGs of patients with pSS, multiple ILs act in concert to shape the immune microenvironment. Dysregulated expression of IL-4 and IL-40 establishes a positive feedback loop that exacerbates tissue injury. IL-4 activates the JAK1/STAT6 pathway to promote Th2 cell differentiation and enhance immunoglobulin G (IgG) secretion by B cells. Driven by aberrant IL-4 production, IL-40 accelerates B lymphocyte activation and demonstrates a positive correlation with ESSDAI scores in pSS patients (128). Excessive secretion of IL-27 promotes activation of the NLRP3 inflammasome, contributing to immune infiltration of the salivary and lacrimal glands through STAT1- and STAT3-dependent signaling, ultimately leading to glandular damage (129). IL-7 and its receptor (IL-7R) are strongly associated with the formation of ectopic lymphoid structures in the MSGs of pSS patients (130). IL-33 further amplifies immune dysregulation by acting synergistically with IL-12 and IL-23 on NK cells to promote IFN-γ production, thereby intensifying local inflammation. Apoptotic SGECs also overexpress IL-33, which activates downstream inflammatory pathways. Elevated IL-33 expression has been proposed as a signal of autoimmune dysregulation and correlates clinically with ocular disease severity in pSS patients (131).

In PBC patients, deficiency of IL-2, an important growth factor that maintains the development of Treg cells, within hepatic tissues suppresses Treg function, thereby aggravating lymphocyte imbalance and promoting aberrant immune responses (132). IL-12 plays a pivotal role in the onset of early biliary inflammation by enhancing IFN-γ production in Th1 cells through the STAT4 signaling pathway, ultimately contributing to hepatic pathology (133). Within the unique immune microenvironment of the PBC liver, the function of interleukins is profoundly altered. Aberrant secretion of IL-37 further amplifies IFN-γ production and promotes the induction of the NK cell–attracting chemokine CCL5. This process, in turn, increases the intrahepatic accumulation of NK and NKT cells, driving persistent inflammatory infiltration and tissue injury (134).

Serum interleukin levels in patients with pSS and PBC represent critical determinants of tissue immune infiltration. In pSS, elevated serum concentrations of IL-6 and IL-9 activate the JAK2/STAT3 signaling pathway, promoting the differentiation and survival of CD4^+^ T cells into Th17 subsets and enhancing IL-17 secretion. These processes establish an inflammatory microenvironment that exacerbates local immune dysregulation and tissue inflammation (135, 136). In PBC, reduced IL-35 expression in the peripheral circulation is associated with heightened T cell-mediated inflammation and cytotoxicity, contributing to progressive abnormalities in liver function tests. Conversely, increased IL-35 expression within tissues impairs Treg activity, indirectly amplifies Th17-mediated immune responses, and accelerates biliary epithelial damage and hepatic fibrogenesis (137).

In patients with pSS and PBC, multiple ILs contribute to abnormal lymphocyte differentiation and distribution within glands and tissues through shared mechanisms, thereby impairing secretory function. IL-21 promotes Th17 differentiation and, together with IL-23, drives lymphocyte expansion. In addition, IL-21 enhances BAFF receptor signaling in B cells or acts directly on B cells to facilitate antibody production. It also induces epithelial cell apoptosis through the Fas/FasL and perforin/granzyme B pathways. IL-23 further supports memory T-cell differentiation and sustains the survival and effector function of Th17 cells. Several interleukins converge on the upregulation of IL-17, primarily through expansion of the Th17 cell population. IL-17 activates both the canonical TGF-β1/Smad2/3 pathway and the non-canonical TGF-β1/Erk1/2 pathway, thereby intensifying tissue inflammation and fibrosis. Elevated IL-17 and IL-23 levels have been detected in the peripheral blood and tissues of patients with pSS and PBC, where they act in a positive feedback loop to amplify inflammation and tissue damage. Clinically, monitoring IL-17 and IL-23 levels may provide value for the early diagnosis of pSS and for staging disease progression in PBC (138–140).

#### 5.3.2 Interferon

IFNs regulate immune responses through the JAK/STAT signaling pathway and other signaling cascades, playing pivotal roles in both innate and adaptive immunity. IFN-α disrupts immune tolerance and induces epithelial cell apoptosis by upregulating MHC class I and II expression and activating antigen-presenting cells. It also promotes lymphocyte proliferation and activation, thereby enhancing autoantibody production. IFN-β shares downstream signaling with IFN-α and exerts primarily anti-inflammatory and anti-proliferative effects (141). IFN-γ enhances innate immune responses by activating the JAK/STAT and NF-κB pathways, drives Tfh cell differentiation and CD8^+^ T cell activation, and contributes to the formation of eGCs and the induction of pathogenic autoantibodies (142).

In patients with pSS, overexpression of IFN-I exacerbates the generation of ROS, induces intracellular oxidative stress, and disrupts normal cellular metabolism. IFN-I further activates key inflammatory pathways, including NF-κB and the NLRP3 inflammasome, thereby amplifying tissue inflammation. Under hypoxic conditions, excessive IFN-I aggravates mitochondrial damage, exacerbates hypoxia, and promotes aberrant apoptotic processes (143). IFN-γ promotes epithelial cell pyroptosis and apoptosis by activating the JAK/STAT1 pathway and upregulating MHC class II expression. In addition, IFN-γ inhibits the cystine/glutamate antiporter system Xc^-^, triggering ferroptosis in SGECs and ultimately impairing MSGs’ function (144).

The mRNA expression levels of IFNs and TLRs are significantly elevated in PBC patients and are distributed across distinct hepatic regions and cell types (145). Enhanced TLR signaling further stimulates IFN- I production, thereby sustaining chronic inflammation and exacerbating autoimmunity. In murine models of PBC, pharmacologic inhibition of the JAK1/2 pathway effectively reduced IFN-γ levels, alleviated portal inflammation and bile duct injury, and decreased CD4^+^ and CD8^+^ T cell infiltration in both the spleen and liver (142). Moreover, female cells exhibit markedly higher IFN-γ production than male cells, highlighting IFN as a key contributor to the sex bias observed in PBC pathogenesis (146).

In pSS patients, peripheral blood IFN-I expression correlates strongly with the production of anti-SSA and anti-SSB antibodies, and is particularly elevated in individuals harboring both anti-Ro52 and anti-Ro60 subtypes (147). In PBC, IFN promotes the generation of sp100 autoantibodies, increases serum AMA antibody levels in PBC murine models, and stimulates CD8^+^ T cell responses that mediate bile duct epithelial injury (148, 149). Thus, elevated IFN expression in both pSS and PBC not only drives abnormal epithelial apoptosis and tissue damage but also exacerbates autoimmunity by facilitating pathogenic autoantibody production.

#### 5.3.3 Tumor necrosis factor

TNF-α, primarily produced by macrophages, plays a central role in orchestrating inflammatory and oxidative processes. It is also implicated in the pathogenesis of chronic hepatic inflammation and fibrosis and is closely linked to BECs injury. In pSS patients, elevated TNF-α levels enhance B-cell activation, thereby promoting the production of IgG, anti-SSA, and anti-SSB antibodies (150). In addition, TNF-α activates the NF-κB signaling pathway, inducing epithelial cell apoptosis and amplifying inflammatory responses. In PBC mouse models, elevated levels of TNF-α or IFN-γ disrupt the barrier function of the tight junction-associated protein 7H6 through NF-κB activation, leading to increased paracellular permeability of cholangiocytes and further disruption of bile duct integrity (151).

B cell activating factor (BAFF), also known as B lymphocyte stimulator (BLyS), is a critical regulator of B cell survival and maturation. By engaging the BAFF receptor (BAFF-R) on B cells it governs B cell differentiation and immune tolerance. In cooperation with a proliferation-inducing ligand (APRIL), BAFF promotes B cell survival, proliferation, and activation, thereby maintaining peripheral B cell homeostasis, sustaining the B cell pool, and supporting humoral immunity. BAFF constitutes a central pathogenic mediator in both pSS and PBC, with its levels closely correlated with autoantibody and immunoglobulin production. In pSS, serum BAFF and APRIL levels are significantly elevated, driving B cell activation, increasing serum IgG secretion, and promoting the generation of anti-SSA and anti-SSB antibodies, thereby intensifying autoimmune responses (152). In addition, BAFF expression is markedly upregulated within inflammatory cells infiltrating the MSGs of pSS patients, where it promotes focal lymphocyte aggregation and eGCs formation, ultimately elevating the risk of lymphoma development (153, 154). In PBC, elevated serum BAFF levels stimulate the production of IgM, IgG, and AMA. It also indirectly contributes to Tregs’ apoptosis and functional impairment, thereby altering the immune microenvironment of biliary epithelial cells (152, 155, 156). Excessive BAFF further aggravates hepatic injury by elevating aminotransferase and bilirubin levels, sustaining hepatocyte necroinflammation, exacerbating cholestasis, and promoting progressive liver fibrosis (157). Collectively, aberrant B cell activation serves as a key mechanistic link between pSS and PBC, with elevated BAFF levels playing a pivotal role in the shared pathogenesis of these two autoimmune diseases.

#### 5.3.4 Chemokines

Aberrant chemokine expression plays a critical role in the pathogenesis of autoimmune responses. Chemokines produced by SGECs and BECs selectively orchestrate the recruitment and homing of distinct lymphocyte subsets, thereby facilitating inflammatory cell infiltration and activating immune cells such as T cells, macrophages, and NK cells. These processes contribute to tissue inflammation and mediate progressive injury to exocrine glands and bile ducts. In patients with pSS and PBC, chemokine levels are elevated to varying degrees across multiple tissues and organs. The specific functions and clinical implications of chemokines involved in immune cell recruitment in pSS and PBC are summarized in **Table 2**.

**Table 2:** pSS and PBC-associated chemokines and their functions and clinical manifestations.

Chemokines	Receptors	Functions	Clinical manifestations
pSS			
CXCL13	CXCR5	CXCL13 is the primary chemokine directing mature B-cell migration and is essential for the formation of ectopic germinal centers.	Elevated CXCL13 expression has been associated with increased production of anti-SSA and anti-SSB antibodies, heightened disease activity, and higher histopathological grading in labial salivary glands. In addition, CXCL13 promotes eGCs development within minor salivary glands and has been implicated in the increased risk of lymphoma (158, 159).
CCL25	CCR9	CCL25 facilitates T-cell maturation and chemotaxis, maintains mucosal immune homeostasis, and contributes to the establishment of immune tolerance.	Upregulated expression of CCR9 and its ligand CCL25 has been observed in disease-associated pathological contexts (160).
CCL19	CCR7	CCL19 contributes to the formation of eGCs.	Its expression is markedly elevated in minor salivary glands and shows strong correlations with anti-SSA antibody positivity and increased serum IgG levels (161).
PBC			
CCL28	CCR10	CCL28 mediates lymphocyte chemotaxis toward epithelial tissues and plays an essential role in mucosal immune homing.	Both CCL28 and its receptor CCR10 are strongly expressed in the bile ducts and portal vein endothelial cells of patients with PBC (162).
CCL3/4/5	CCR1/3/5	CCL3, CCL4, and CCL5 promote T cell expansion and the recruitment of innate immune cells, thereby amplifying inflammatory responses and driving the progression of hepatic fibrosis.	These chemokines are strongly expressed in hepatic Kupffer cells, T cells, NK cells, and epithelial cells (163).
CXCL16	CXCR6	CXCL16 is broadly expressed on the surface of various immune cells, where it facilitates lymphocyte recruitment to the bile ducts and enhances antigen presentation.	CXCL16 promotes the recruitment of T cells to the liver and their infiltration into inflamed portal tracts, a process associated with elevated markers of cholestasis (164).
CCL11/24/26	CCR3	CCL11, CCL24, and CCL26 induce eosinophil chemotaxis.	Serum levels of CCL11, CCL24, and CCL26 are elevated in patients with PBC, with CCL11 and CCL26 specifically associated with fibrosis progression (165).
pSS and PBC			
CX3CL1	CX3CR1	CX3CL1 mediates the recruitment of intraepithelial lymphocytes and promotes the migration of macrophages and NK cells.	In patients with pSS, CX3CL1 levels are significantly elevated in minor salivary glands, lacrimal glands, and serum (166).
			In PBC, CX3CL1 is specifically upregulated and is associated with enhanced lymphocyte infiltration and exacerbated inflammatory responses within the bile ducts (167).
CXCL12	CXCR4	CXCL12, a B-cell pre-growth factor, promotes lymphangiogenesis and facilitates B-cell homing.	In patients with pSS, CXCL12 expression is upregulated in lymphocyte-infiltrated regions of the salivary and lacrimal glands and shows a strong correlation with lymphoma development (168).
			The CXCL12/CXCR4 axis also contributes to hepatic fibrosis in PBC by enhancing B-cell infiltration and modulating hepatic stellate cell activity (169).
CXCL9/10/11	CXCR3	CXCL9, CXCL10, and CXCL11 activate and recruit Th1 cells, thereby promoting type 1 adaptive immune responses.	In patients with pSS, these chemokines are significantly upregulated in (MSGs, where their expression strongly correlates with lymphocytic focus scores and glandular dysfunction (170).
			Moreover, the frequency of CXCR3-expressing cells is markedly increased in both the liver and peripheral blood (171).
CCL21	CCR7	CCL21 initiates immune responses, sustains immune tolerance, recruits and activates T cells, and promotes B cell expansion and survival.	In pSS, elevated CCL21 expression is associated with increased ESR, higher serum IgG and RF levels, and elevated anti-SSA/SSB antibody titers, and is considered an important factor contributing to lymphoma development (172).
			In PBC, CCL21 is prominently expressed in lymphoid aggregates within portal tracts (173).

pSS, primary Sjogren’s syndrome; CXCL, chemokine C-X-C motif ligand; CXCR, C-X-C chemokine receptor; eGCs, ectopic germinal centers; MSGs, minor salivary glands; CCL, chemoattractant chemokine ligand; CCR, C-C chemokine receptor; IgG, immunoglobulin G; PBC, primary biliary cholangitis; NK cells, natural killer cells; ESR, erythrocyte sedimentation rate.

CX3CL1 is a chemokine with dual functions in lymphocyte recruitment and cell adhesion. Acting through its receptor CX3CR1, it mediates the recruitment of intraepithelial lymphocytes and facilitates the migration and accumulation of macrophages and NK cells. CX3CL1 is upregulated to varying degrees in patients with both pSS and PBC. In pSS, CX3CL1 and CX3CR1 are primarily localized in salivary and lacrimal glands, where they drive immune cell infiltration and are associated with eGCs formation. Experimental blockade of the CX3CL1/CX3CR1 axis in SS mouse models reduced M1 macrophage infiltration, alleviated SG inflammation, and restored glandular function (174). In PBC, CX3CL1 upregulation mainly occurs in senescent BECs, where it facilitates the migration and accumulation of CX3CR1^+^ lymphocytes around bile ducts. In turn, this process exacerbates lymphocytic bile duct inflammation via activation of the NF-κB signaling pathway (167).

CXCL12, a B-cell growth factor, promotes lymphangiogenesis and regulates the activation, adhesion, and homing of CXCR4^+^ cells. In patients with pSS, CXCL12^+^ cells are highly expressed in B-cell-infiltrated salivary and lacrimal glands, particularly within malignant B cells, and this upregulation is associated with an increased risk of lymphoma (168). In PBC, CXCL12 activates B cells through CXCR4 signaling and modulates the production of pro-fibrotic cytokines in the liver. Experimental knockdown of CXCL12 or pharmacologic blockade of the CXCL12/CXCR4 axis reduces hepatic B-cell infiltration and mitigates liver fibrosis (169).

The CXCL9/CXCL10/CXCL11-CXCR3 axis promotes Th1 cell activation and induces IFN-γ expression, thereby promoting T lymphocyte migration to inflammatory sites. In the MSGs of patients with pSS, expression of CXCL9, CXCL10, and CXCL11 is markedly upregulated and strongly correlates with lymphocyte focus scores and the severity of glandular dysfunction (170). In patients with PBC, serum levels of CXCL9 and CXCL10 are significantly elevated, accompanied by an increased frequency of CXCR3^+^ cells in both the liver and peripheral blood, which enhances effector T cell recruitment to affected tissues (171). In PBC animal models, suppression of CXCL10 expression attenuates hepatic inflammation and fibrosis, while CXCR3 deficiency mitigates inflammatory progression (175). These findings indicate that targeting the CXCL9/CXCL10/CXCL11-CXCR3 axis may represent a promising therapeutic strategy for PBC.

The CCL21/CCR7 axis regulates the initiation of immune responses and maintains immune tolerance. This pathway mediates the chemotactic migration of lymphocytes into MSGs and represents a critical factor in lymphoma development among pSS patients. In PBC, CCL21 is strongly expressed within lymphoid aggregates of the portal tracts (173, 176). By enhancing lymphocyte activation and proliferation around damaged bile ducts and glands, the CCL21/CCR7 axis markedly increases the extent of lymphocytic infiltration. In pSS, elevated CCL21 levels are associated with abnormal ESR and RF, while activated B cells produce higher levels of IgG, anti-SSA, and anti-SSB antibodies, collectively resulting in elevated serum autoantibody titers (168, 172).

## 6 Targeted drug similarities

In clinical practice, the management of patients with both pSS and PBC requires a comprehensive evaluation of disease status, with personalized therapeutic strategies tailored to disease activity and the extent of systemic involvement.

Current clinical approaches to pSS primarily target local manifestations such as xerostomia and keratoconjunctivitis sicca. Localized therapies are frequently employed to alleviate symptoms, reduce tissue injury, and mitigate systemic dysfunction. In patients with parotid gland enlargement or severe organ involvement, corticosteroids (e.g., prednisone, methylprednisolone) and immunosuppressive agents (e.g., cyclophosphamide, hydroxychloroquine) can effectively suppress inflammatory responses, though regular clinical monitoring is essential throughout treatment. Traditional Chinese medicine provides a multi-targeted therapeutic strategy, acting through diverse pathways. Tripterygium glycosides, for example, possess anti-inflammatory and immunomodulatory properties, providing multiple therapeutic targets and demonstrating promising benefits in pSS management. However, their long-term efficacy and safety remain to be fully established (177).

The therapeutic goals for PBC are to alleviate cholestatic symptoms, control hepatic inflammation, delay the progression of liver fibrosis, and prevent complications of end-stage liver disease. Ursodeoxycholic acid (UDCA), the current first-line therapy, promotes bile flow by facilitating anion exchange and bile alkalinization, thereby reducing bile acid toxicity, intrahepatic cholestasis, and bile duct injury. Beyond its choleretic effects, UDCA exerts immunomodulatory effects by stabilizing mitochondrial membranes, suppressing IFN-γ production, and improving the hepatic immune microenvironment, which collectively attenuate autoimmune-mediated damage. Nevertheless, approximately 40% of patients exhibit an insufficient response to UDCA, with its therapeutic efficacy markedly reduced in advanced stages of PBC (178).

**Figure 2:**
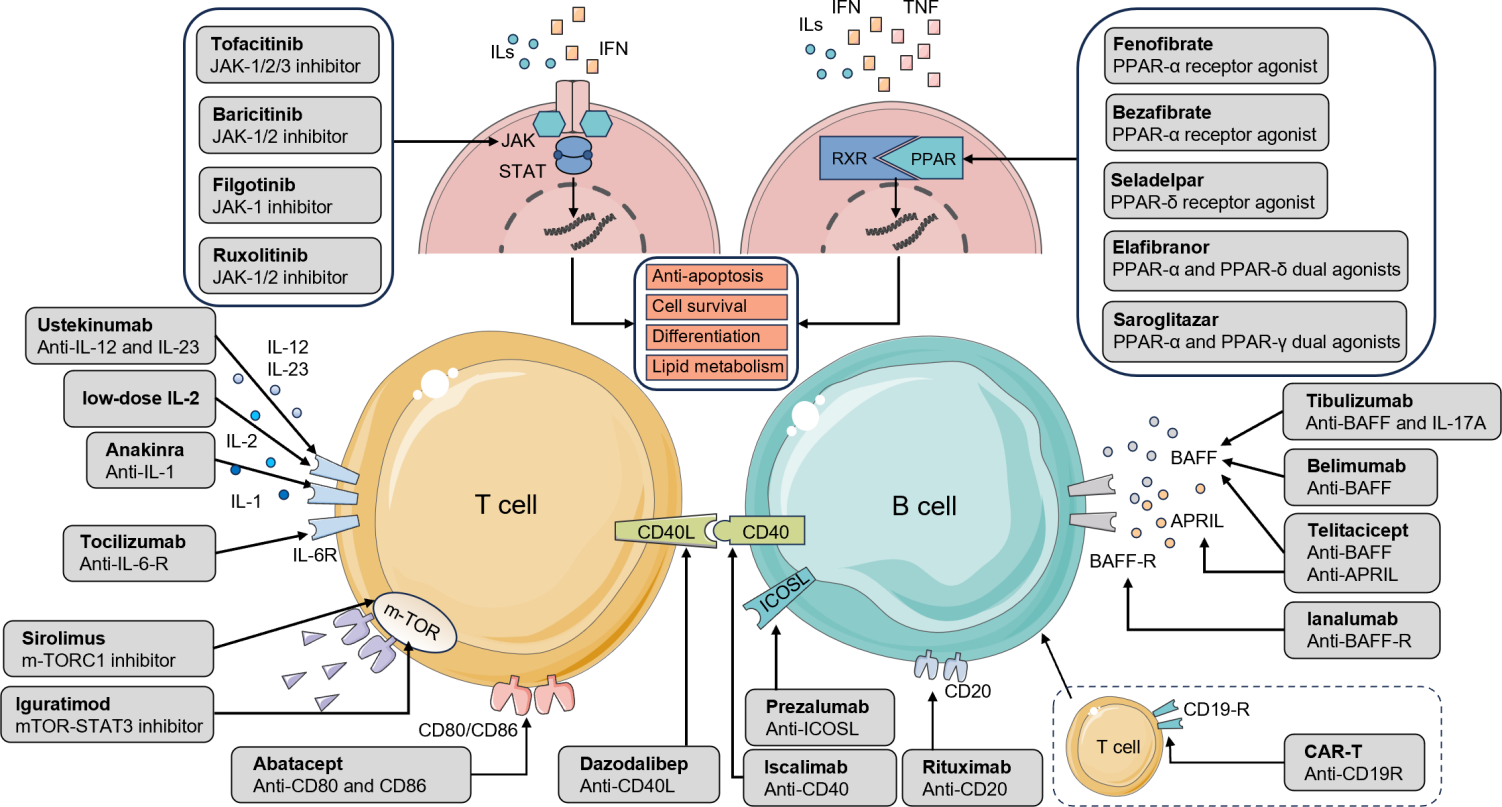
Drugs targeting major pathways and receptors in the pathophysiology of pSS and PBC. The interaction between T and B cells is a crucial event in the pathogenesis of pSS and PBC. Therapeutic agents that act on lymphocyte surface receptors or related signaling pathways can attenuate immune-mediated attacks against self-antigens, thereby providing clinical benefit. Cytokines such as ILs, IFNs, and TNFs activate the JAK/STAT and PPAR pathways, driving lymphocyte survival, differentiation, and antibody production. JAK inhibitors and PPAR agonists suppress aberrant lymphocyte proliferation and differentiation while improving tissue lipid metabolism. The mTOR pathway regulates protein synthesis, cellular metabolism, and growth; its inhibition suppresses Tfh cell differentiation, restores the Tfr/Tfh balance, and alleviates tissue inflammation. Interleukin inhibitors modulate T-cell subset function, dampen hyperactive immune responses, and restore immune homeostasis, thereby mitigating autoimmune injury in conditions such as pSS and PBC. Additional therapeutic targets include co-stimulatory molecules. By blocking CD80/CD86 binding to CD28 on T cells, agents prevent co-stimulatory signaling, thereby limiting T-cell activation, proliferation, and cytokine secretion. The CD40/CD40L axis contributes to sustained immune activation and antibody production, processes central to germinal center formation. Targeting ICOSL reduces both B-cell and Th17 cell activation. BAFF and APRIL act synergistically to promote B-cell survival, proliferation, and activation, regulate peripheral B-cell homeostasis, and maintain humoral immunity. Inhibition of BAFF signaling effectively suppresses B-cell activation and autoantibody production. Anti-CD20 therapies enhance cytotoxic responses against B lymphocytes, resulting in their depletion. Finally, CAR-T cell therapy, by engineering autologous T cells to recognize and eliminate B cells, represents an innovative approach to reducing B-cell numbers and suppressing their pathogenic activity. pSS, primary Sjogren’s syndrome; PBC, primary biliary cholangitis; JAK/STAT, Janus kinase-signal transducer and activator of transcription; PPAR, peroxisome proliferator-activated receptor; mTOR, mechanistic target of rapamycin; Tfr, follicular regulatory T cells; Tfh, T follicular helper; CD, cluster of differentiation; ICOSL, inducible T-cell co-stimulator ligand; Th, T helper; BAFF, B-cell activating factor; APRIL, a proliferation-inducing ligand; CAR-T, Chimeric Antigen Receptor T-cell.

Localized therapies for pSS and PBC provide only symptomatic relief of glandular dysfunction and fail to address the underlying fundamental immune dysregulation underlying these diseases, including abnormal immune cell proliferation and differentiation. In recent years, extensive efforts have been directed toward targeting immune regulatory pathways and modulating both the quantity and function of immune cells, yielding significant progress. In this review, we systematically classify and summarize the major targeted therapeutic agents and their related signaling pathways in the clinical management of pSS and PBC, with detailed information presented in **Table 3** and **Figure 2**.

**Table 3:** Clinical Therapeutic Targets, Drugs, and Mechanisms for pSS and PBC.

Evaluated drug	Diseases	Phase	Drug description	Results
Targeting the JAK pathway				
Tofacitinib	pSS	III	JAK-1/2/3 inhibitor	Tofacitinib has been shown to improve both objective signs and subjective symptoms of dry eye disease, while also demonstrating a favorable safety and tolerability profile (179).
	PBC	Preclinical trials		An international genome-wide meta-analysis suggested that tofacitinib may exert pharmacological effects in PBC; however, no clinical trials have yet been conducted to confirm its therapeutic efficacy in this context (180).
Filgotinib	pSS	II	JAK-1 inhibitor	Filgotinib improved total ESSDAI scores, significantly reduced RF and IFN activity, and decreased immunoglobulin production (181); however, the phase II randomized controlled trial did not meet its primary endpoint.
Baricitinib	pSS	II	JAK-1/2 inhibitor	In a study, baricitinib reduced ESSDAI scores, improved arthritis and cutaneous manifestations, and decreased both IgG and ESR levels (182).
	PBC	II		Baricitinib treatment led to reductions in serum ALP levels, alleviation of pruritus and depressive symptoms, and decreases in histological markers of hepatic inflammation and fibrosis (183).
Ruxolitinib	PBC	Preclinical trials	JAK-1/2 inhibitor	Ruxolitinib improved hepatic histopathology, reduced pro-inflammatory cytokine levels, and suppressed GC formation (142).
Targeting the mTOR pathway				
Sirolimus	pSS	II	mTORC1 inhibitor	Sirolimus reduced immunoglobulin production and alleviated inflammatory damage (184).
	PBC	Preclinical trials		Clinical and preclinical studies of this target and drug in PBC remain limited (185).
Iguratimod	pSS	IV	Akt/mTOR/STAT3 inhibitor	Iguratimod improved inflammatory markers (ESR, IgG, and RF levels), restored salivary and lacrimal gland secretory function, and reduced ESSDAI and ESSPRI scores in patients with pSS (186).
Targeting interleukins				
Ustekinumab	pSS	I	Monoclonal antibodies to IL-12 and IL-23	Ustekinumab improved joint manifestations in patients with pSS and concomitant psoriasis (187).
	PBC	II		Ustekinumab reduced serum ALP levels in patients with PBC (188).
Low-dose IL-2 therapy	pSS	II	Recombinant human IL-2	Low-dose IL-2 therapy decreased pro-inflammatory cytokine levels, restored Treg numbers and the Th17/Treg ratio in peripheral blood, and improved platelet and leukocyte counts, although it did not reduce autoantibody production (189).
	PBC	II		The optimal dosage and safety profile of Low-dose IL-2 therapy remain under investigation.
Anakinra	pSS	II	Anti-IL-1	Anakinra did not significantly improve fatigue symptoms (190).
Tocilizumab	pSS	II	Anti-IL-6R	Tocilizumab did not improve disease activity scores (191).
Targeting PPAR agonists				
Fenofibrate	pSS	Preclinical trials	PPAR-α agonist	Fenofibrate achieved favorable outcomes in preclinical studies, but its efficacy has not yet been extensively validated in large-scale clinical trials (192).
	PBC	III		Fenofibrate improved serum ALP and IgM levels, reduced intracellular bile acid concentrations in hepatocytes and hepatic biochemical parameters, and enhanced transplant-free survival (193).
Bezafibrate	PBC	III	PPAR-α agonist	Bezafibrate alleviated pruritus, improved liver fibrosis, and reduced relevant biochemical markers. However, treatment was associated with elevated serum creatinine levels and adverse events such as muscle and joint pain (194).
Seladelpar	PBC	III	PPAR-δ agonists	Seladelpar reduced ALP levels and liver biochemical markers, and significantly improved pruritus and fatigue in patients (195, 196).
Elafibranor	PBC	III	PPAR-α and -δ agonists	Elafibranor reduced liver biochemical abnormalities and significantly decreased serum inflammatory and autoantibody marker levels. However, the incidence of adverse events was relatively high (197, 198).
Saroglitazar	PBC	III	PPAR-α and PPAR-γ agonists	Saroglitazar reduced biochemical markers of cholestasis in patients with PBC (199).
Targeting T-lymphocytes				
Abatacept	pSS	III	CTLA4-Fc fusion protein	Abatacept improved disease activity, laboratory parameters, and patient-reported fatigue, accompanied by a reduction in serum anti-SSA and anti-SSB antibody levels (200, 201).
	PBC	IV		Abatacept reduced inflammation surrounding portal veins and bile ducts, as evidenced by liver biopsy findings, and was associated with decreased liver biochemical markers (202).
Targeting Lymphocyte Stimulating Factor				
Belimumab	pSS	II	Monoclonal antibodies to BAFF	Belimumab improved the disease activity index in pSS patients, alleviated subjective symptoms including dryness, pain, and fatigue, reduced parotid gland swelling and lymphadenopathy, and led to a sustained decrease in B-cell activation markers (203, 204).
	PBC	III		Belimumab reduced serum IgM levels in PBC patients, although it did not improve the extent of cholestasis (205).
Ianalumab	pSS	III	Anti-BAFF receptor monoclonal antibody	Ianalumab demonstrated improvement in ESSDAI scores and a reduction in salivary flow rate (206).
Remibrutinib	pSS	II	BTK receptor inhibitors	Remibrutinib significantly improved total ESSDAI scores, but did not alleviate symptoms of dryness, pain, fatigue, or restore glandular function (207).
Telitacicept	pSS	III	TACI–Fc fusion protein	Telitacicept improved ESSDAI scores and reduced serum autoantibody levels, but did not significantly improve fatigue, pain, or dryness symptoms (208).
Dazodalibep	pSS	II	CD40L Antagonist	Dazodalibep significantly reduced serum CXCL13 and RF levels, with improvements in dryness, fatigue, and pain symptoms, as well as in ESSDAI and ESSPRI scores (209).
Iscalimab	pSS	II	Anti-CD40 monoclonal antibody	Iscalimab improved MSG’s function and led to reductions in both ESSDAI and ESSPRI scores (210).
Tibulizumab	pSS	I	Bispecific dual antagonist antibodies to BAFF and IL-17A.	Tibulizumab clinical and preclinical investigations remain limited.
Prezalumab	pSS	II	Anti-ICOS	Prezalumab did not demonstrate significant improvement in disease activity (RCT02334306).
Targeting B lymphocytes				
Rituximab	pSS	III	Anti-CD20 monoclonal B-cell depletion antibody	Rituximab relieved early-stage symptoms of pSS, including fatigue, dryness, and pain (211).
	PBC	II		Rituximab significantly reduced serum ALP levels and plasma concentrations of AMA, IgM, and IgG; however, no notable improvement was observed in liver biochemical parameters (212–214).

pSS, primary Sjogren’s syndrome; PBC, primary biliary cholangitis; JAK, Janus kinase; ESSDAI, EULAR Sjogren’s syndrome disease activity index; IFN, interferon; RCT, randomized controlled trial; ESR, erythrocyte sedimentation rate; ALP, alkaline phosphatase; GC, germinal center; RAPA, peroxisome proliferator-activated receptor; mTORC1, mechanistic target of rapamycin complex 1; IgG/M, immunoglobulin G/M; RF, rheumatoid factor; IL, interleukin; Th, T helper; Tregs, regulatory T cells; PPAR, peroxisome proliferator-activated receptor; CTLA4-Fc fusion protein, cytotoxic T-lymphocyte antigen 4-Fc fusion protein; BAFF, B cell activating factor; BTK, Bruton’s tyrosine kinase; TACI-Fc fusion protein, transmembrane activator and CAML interactor-Fc fusion protein; CD, cluster of differentiation; CXCL, chemokine C-X-C motif ligand; ESSPRI, EULAR Sjogren’s syndrome patient reported index; ICOS, inducible T-cell co-stimulator ligand; AMA, antimitochondrial antibodies.

### 6.1 Therapy targeted pathways and receptors

#### 6.1.1 Targeting the JAK/STAT signaling pathway

The JAK/STAT signaling pathway is a critical intracellular cascade of protein interactions. Upon cytokine stimulation, Janus kinases (JAKs) phosphorylate signal transducers and activators of transcription (STATs), leading to their dimerization and subsequent translocation into the nucleus, where they regulate the expression of specific target genes. In patients with pSS and PBC, aberrant activation of the JAK/STAT pathway, particularly through IFN-I-mediated signaling, contributes to the heightened activation of both T and B lymphocytes. Targeted therapies aimed at inhibiting this pathway have shown encouraging therapeutic efficacy in the treatment of these diseases, positioning the JAK/STAT axis as a promising target for immunomodulatory intervention.

Baricitinib, a selective JAK1/2 inhibitor, exerts immunomodulatory effects by inhibiting JAK enzymatic activity, thereby reducing immune cell proliferation and activation, as well as autoantibody production. Exerts immunomodulatory effects by inhibiting JAK enzymatic activity, thereby reducing immune cell proliferation and activation, as well as autoantibody production. These mechanisms contribute to the therapeutic improvement of autoimmune diseases. In pSS, a preliminary study indicates that baricitinib can inhibit IFN-γ and CXCL10 expression in MSGs, significantly decrease ESSDAI scores, and alleviate arthritis and cutaneous manifestations. Serum IgG levels and ESR also declined during treatment, with the exception of the 3rd month (182). In PBC, baricitinib treatment is associated with a markedly faster reduction in serum ALP, alongside improvement in pruritus and depressive symptoms. Liver histological analyses further revealed decreased hepatic inflammation and fibrosis, indicating superior efficacy compared with conventional therapies such as UDCA (183). Currently, a phase II randomized, double-blind, placebo-controlled proof-of-concept trial (NCT03742973) is underway to further assess the therapeutic potential of baricitinib in PBC.

#### 6.1.2 Targeting the mTOR signaling pathway

The mechanistic target of rapamycin (mTOR) signaling pathway plays a central role in intracellular signal transduction. By activating mTOR and its upstream and downstream effectors, this pathway regulates protein synthesis and cellular metabolism, thereby governing cell growth and proliferation.

Sirolimus, a potent mTORC1 inhibitor, exerts its immunomodulatory effects by restricting mTORC1 activation, which suppresses the differentiation of Tfh cells and restores the balance between Tfr and Tfh cells. This subsequently attenuates B-cell activation and alleviates tissue inflammation. In patients with pSS, mTOR inhibition effectively suppresses B-cell proliferation and immunoglobulin production, mitigating immune-mediated inflammation and facilitating the restoration of lacrimal gland function (184). In experimental SS models, sirolimus downregulated mTOR pathway activity, reduced the expression of inflammatory cytokines and chemokines, and inhibited Th17 cell differentiation as well as IL-17 secretion. These effects collectively resulted in substantial reductions in SG lymphocyte infiltration and inflammation (215). In zebrafish mutants, hyperactivation of the PI3K/AKT/mTOR pathway in hepatic tissue has been linked to PBC-like lesions, and sirolimus treatment partially ameliorated these pathological features, suggesting a potential therapeutic role (185). However, clinical and preclinical data regarding mTOR as a therapeutic target in PBC remain limited and warrant further investigation.

#### 6.1.3 Targeting the interleukin pathway

Ustekinumab is a monoclonal antibody that targets IL-12 and IL-23 by binding to their shared p40 subunit, thereby preventing cytokine-receptor interactions, suppressing IFN-γ production, and attenuating Th1/Th17 responses. In a proof-of-concept clinical trial for PBC, 28 weeks of ustekinumab therapy led to a moderate reduction in serum ALP levels (187). However, its precise role in modulating the immunopathology of PBC remains to be fully elucidated. Clinically, ustekinumab has also demonstrated significant improvement in joint involvement among psoriasis patients with concomitant pSS. Notably, no pSS-related signs or symptoms were observed during long-term follow-up (188). Furthermore, a pilot study (NCT04093531) showed reductions in both ESSPRI and ESSDAI scores after 24 weeks of treatment. Currently, a Phase I open-label trial is enrolling participants to further assess ustekinumab in pSS, and forthcoming data are expected to provide deeper insights into its therapeutic potential.

Low-dose interleukin-2 (LD-IL-2) therapy holds significant promise in the treatment of pSS and PBC. In SS murine models, LD-IL-2 administration effectively restored the proportion of Tregs in the spleen and cervical lymph nodes (216). Clinical trials in pSS patients demonstrated that LD-IL-2 therapy effectively increases peripheral Treg numbers, rebalances the Th17/Treg ratio, reduces pro-inflammatory cytokine levels, and improves the local immune microenvironment. Additionally, LD-IL-2 therapy has also shown clinical benefits in correcting thrombocytopenia and leukopenia. However, LD-IL-2 failed to reduce autoantibody levels (189). In PBC murine models, LD-IL-2 treatment was found to restore Th17/Treg balance, attenuate inflammatory infiltration in the portal area, and alleviate bile duct injury (217). Despite these encouraging findings, the optimal dosing strategy and safety profile of LD-IL-2 therapy in patients with PBC remain under investigation in ongoing preclinical and early-phase clinical studies.

#### 6.1.4 Targeting the PPAR

Peroxisome proliferator-activated receptors (PPARs) are a class of nuclear receptors that regulate intracellular lipid and energy metabolism, and they also exhibit significant anti-inflammatory properties. The PPAR family includes PPAR-α and PPAR-γ, both of which play critical roles in modulating immune responses in T and B lymphocytes. PPAR-α regulates immune activity by antagonizing NF-κB and IL-1β signaling pathways in T cells, whereas PPAR-γ suppresses T cell proliferation and differentiation by preventing NFAT and NF-κB binding to the IL-2 promoter. In addition, PPAR-γ contributes to the regulation of B cell homeostasis by promoting apoptosis via cytotoxic mechanisms, effectively limiting B cell proliferation. Notably, PPAR-α and PPAR-γ regulate IFN-γ and IL-17 production in T cells in a sex-specific manner. This sexually dimorphic regulation may partially account for the higher prevalence of T cell–mediated autoimmune diseases observed in females (218).

Fenofibrate, a commonly used PPAR-α agonist, regulates lipid metabolism and alleviates chronic inflammation associated with lipid accumulation. Owing to its anti-inflammatory and metabolic effects, fenofibrate has therapeutic potential in various autoimmune diseases. In patients with PBC, fenofibrate has been reported to lower intrahepatic bile acid concentrations, thereby reducing bile acid-induced cytotoxicity toward cholangiocytes. It also improves serum ALP levels and suppresses hepatic and biliary inflammation, while maintaining a favorable clinical safety profile (193, 219). It has also been shown to reduce IgM levels and AMA antibody titers (193, 220). Preclinical studies further demonstrate that fenofibrate effectively suppresses Th1/Th17 responses in SS mouse models, increases both the number and functional activity of Tregs, and ameliorates histopathological features of autoimmune sialadenitis. These encouraging findings highlight its potential as a novel therapeutic approach for pSS (192). However, the clinical application of fenofibrate in pSS remains insufficiently validated, with limited trial data available to confirm its efficacy as a standalone therapy.

### 6.2 Therapeutic pathways for targeting lymphocytes

#### 6.2.1 Targeting T lymphocytes

Abatacept is a humanized fusion protein composed of cytotoxic T-lymphocyte-associated antigen 4 (CTLA-4) linked to the Fc region of human IgG1. By binding to CD80 and CD86 expressed on antigen-presenting cells, it blocks T-cell costimulatory signaling. This mechanism effectively reduces T-cell activation and proliferation, reprograms T-cell function, and ultimately attenuates T-cell-mediated immune response.

In a clinical trial involving patients with RA, abatacept demonstrated the potential to improve salivary and tear secretion, providing a theoretical rationale for its application in the treatment of pSS (221). In a subsequent study of fifteen patients with pSS, abatacept reduced circulating Tfh and Treg cell populations, decreased serum levels of IL-21, CXCL13, and CXCL1, inhibited lymphocyte migration, and attenuated inflammatory infiltration. It also decreased PC numbers and reduced serum anti-SSA and anti-SSB antibody levels, thereby suppressing overall disease activity (200). Clinical investigations in early-stage pSS further showed that abatacept improved disease activity, laboratory parameters, and fatigue; however, its therapeutic benefits appeared less pronounced in individuals with moderate to severe disease activity (201). A single-center phase III trial reported that 48 weeks of abatacept treatment alleviated pSS symptoms, improved dry eye manifestations, significantly reduced disease activity, downregulated T-cell activation markers, and diminished lymphocytic infiltration (222). In contrast, another investigation failed to demonstrate symptomatic improvement in pSS patients (223). These findings highlight the need for additional randomized controlled trials to comprehensively assess the efficacy of abatacept in pSS and to clarify its effects on specific disease manifestations.

Abatacept has also been extensively studied as a potential therapeutic agent for PBC. Preclinical studies demonstrated that it reduced intrahepatic T-cell infiltration and alleviated BECs injury, although it failed to decrease serum levels of autoantibodies, particularly AMA (224). In clinical settings, combination therapy with sulfasalazine and abatacept normalized ALP and γ-GT levels in PBC patients, accompanied by histological evidence of reduced portal and periductal inflammation on liver biopsy (202). However, in a randomized controlled trial involving 22 PBC patients, only one participant (6.3%) achieved ALP normalization after 24 weeks of abatacept monotherapy, while no significant clinical improvements were observed in the remaining subjects (225). Mechanistically, abatacept acts primarily by inhibiting T-cell co-stimulatory signaling, but it does not effectively suppress terminally differentiated T or B cells, particularly CD8^+^ T cells. Consequently, neither preclinical models nor clinical trials have demonstrated consistent reductions in autoantibody production. These findings suggest that the efficacy of abatacept in PBC remains uncertain and warrants further clinical validation through larger, well-designed studies.

#### 6.2.2 Targeting lymphocyte activating factor

B-cell activation constitutes a pivotal mechanism in the shared immunopathogenesis of pSS and PBC. Elevated BAFF levels are strongly associated with both diseases, sustaining autoantibody production by promoting plasma cell survival.

Belimumab, a monoclonal antibody that targets BAFF, exerts its immunomodulatory effects by inhibiting B-cell maturation and reducing B-cell activation. Clinical studies have shown that belimumab significantly improves the pSS disease activity index, alleviates subjective symptoms such as dryness, pain, and fatigue, and reduces parotid gland swelling and lymphadenopathy. These clinical improvements are accompanied by a sustained decline in biomarkers associated with B-cell activation (203, 204). In a reported PBC case, belimumab has been investigated in limited clinical settings. In one case report, treatment with belimumab successfully reduced serum IgM levels; however, no improvement was observed in the degree of cholestasis or hepatic fibrosis (205). Although BAFF-targeting therapies such as belimumab show considerable promise for the management of PBC, no agents have yet received regulatory approval for this indication. Further randomized controlled trials are required to establish their efficacy and define their role in clinical practice.

Telitacicept, a novel TACI-Fc fusion protein, suppresses B-cell development and maturation by blocking both BAFF and APRIL signaling pathways. By preventing the differentiation of mature B cells into antibody-secreting PCs, it effectively reduces the secretion of pathogenic autoantibodies. In a multicenter, randomized, double-blind, placebo-controlled phase II trial in pSS (NCT04078386), telitacicept significantly improved ESSDAI scores and decreased serum autoantibody levels at weeks 12 and 24. However, despite these objective improvements in disease activity, patient-reported symptoms such as fatigue, pain, and dryness did not show meaningful relief (208). More recently, telitacicept has demonstrated encouraging results in additional clinical studies for pSS and has progressed to a phase III clinical trial (CTR20223429). Notably, in a case involving a patient with pSS and comorbid PBC who was unresponsive to combined corticosteroid and immunosuppressive therapy, telitacicept treatment resulted in marked improvements in liver function parameters and reduced corticosteroid dependence. Collectively, these findings highlight telitacicept as a promising therapeutic candidate for both pSS and PBC.

#### 6.2.3 Targeting B-lymphocytes

Rituximab is a monoclonal antibody targeting the CD20 surface antigen expressed on pre-B and mature B lymphocytes. By binding to CD20, rituximab initiates B-cell depletion through multiple cytotoxic mechanisms, including complement-dependent cytotoxicity and antibody-dependent cell-mediated cytotoxicity, leading to the depletion of peripheral B lymphocytes.

Early clinical trials demonstrated that rituximab effectively alleviates certain early symptoms of pSS, such as fatigue and parotid gland enlargement. However, its effects on pSS symptoms remain inconsistent, and its long-term efficacy is still uncertain (211). More recent studies have shown that repeated rituximab infusions and extended treatment duration can effectively reduce peripheral immune activation, limit B-cell recruitment into the MSGs, and inhibit the formation of eGCs (226). Despite successful B-cell depletion, immunoglobulin-producing cells persist in the MSGs of pSS patients following rituximab treatment, and B-cell populations remain detectable in the glands (227). Recognizing the limitations of rituximab monotherapy, combination strategies, such as co-administration of rituximab (anti-CD20) with belimumab (anti-BAFF), are currently under investigation. This combined approach has achieved profound and durable depletion of CD20^+^ and CD19^+^ B-cell subsets in both MSGs and peripheral blood, representing a potentially more effective therapeutic strategy (228).

Rituximab is the first biologic agent introduced for the treatment of PBC. In a randomized trial involving 57 patients with PBC, three months of rituximab infusion led to a significant reduction in serum ALP levels; however, no meaningful improvement in fatigue was observed (212). Treatment also lowered plasma IgM and IgG concentrations and reduced AMA secretion, although overall improvements in hepatic biochemical parameters remained modest. Importantly, these effects were transient, suggesting that repeated administration may be necessary to sustain therapeutic efficacy (213, 214). In preclinical PBC models, combination therapy targeting both BAFF and CD20 significantly reduced B-cell populations in peripheral blood and hepatic tissues, while attenuating portal inflammation and bile duct injury-highlighting its potential therapeutic value (229). Overall, rituximab demonstrates therapeutic potential in both pSS and PBC, particularly in combination regimens. Nevertheless, B-cell depletion alone may be insufficient to restore glandular function, and the durability and clinical applicability of rituximab therapy remain to be further validated.

With the increasing understanding of pSS pathogenesis, a growing number of therapeutic agents targeting lymphocyte subsets and cell surface receptors are under active investigation. Ianalumab, a dual B-cell depleting and BAFF receptor-blocking antibody; epratuzumab, an inhibitor of B-cell activation; and iscalimab, a monoclonal antibody that suppresses CD40-CD40L-mediated co-stimulatory signaling between T and B cells, have all demonstrated encouraging therapeutic effects in clinical studies. In addition, chimeric antigen receptor T (CAR-T) cell therapy, an emerging immunotherapeutic approach, has been applied in refractory pSS. This strategy involves engineering autologous T cells to express chimeric antigen receptors directed against CD19, enabling selective recognition and elimination of B cells. In pSS, CAR-T therapy primarily functions to deplete B cells and restore immune homeostasis. Looking forward, CAR-T therapy is expected to be further explored in PBC, where it may provide a more targeted therapeutic option tailored to specific patient populations and disease phenotypes, with the potential to correct autoimmune dysregulation at its root.

## 7 Conclusions

Both pSS and PBC are autoimmune diseases that share striking similarities in their underlying pathogenic mechanisms. Genetic predisposition, X chromosome-linked factors, sex hormones, viral infections, and environmental triggers collectively contribute to the initiation or amplification of autoimmune responses, ultimately leading to impaired glandular and liver tissue.

From an immunological perspective, pSS and PBC are closely interconnected, displaying common autoantibody profiles, including ACA. Histologically, both conditions are characterized by comparable patterns of lymphocytic aggregation and infiltration surrounding glands and ducts.

In the tissue immune microenvironment, both pSS and PBC display similar patterns of epithelial injury and lymphocytic infiltration. Hypoxia and senescence of epithelial cells promote aberrant cell death and facilitate their role as autoantigen-presenting cells, while dysregulated fibroblast activation accelerates inflammation and fibrosis. Disbalance between T and B lymphocytes, particularly the excessive activation of B cells, emerges as a pivotal driver of autoimmunity. Elevated levels of IL-17 and IL-23 enhance Th17 cell function and abnormal B-cell activation, whereas increased IFN and TNF expression further stimulate antigen-presenting cells and B lymphocytes, amplifying autoantibody production. Additionally, upregulation of chemokines such as CXCL9, CXCL10, CXCL11, CXCL12, and CCL21 promotes lymphocyte infiltration into glandular tissues and fosters the formation of ectopic germinal centers. Collectively, these immunopathological processes represent key shared mechanisms driving disease progression in both conditions.

Therapeutically, the shared pathogenic mechanisms of pSS and PBC provide opportunities for common treatment strategies. Targeted agents against the JAK/STAT pathway (tofacitinib and baricitinib), the mTOR signaling pathway (sirolimus), the IL-12/23 receptor (ustekinumab), and PPAR agonists (fenofibrate) have all demonstrated favorable clinical outcomes. Lymphocyte-directed approaches, including abatacept, which modulates T-cell activation, and rituximab, which depletes B cells, have also shown promising efficacy; however, further clinical data are needed to confirm their long-term benefits. BAFF-driven B-cell activation plays a pivotal role in both diseases. Inhibitors of BAFF, such as telitacicept and belimumab, have significantly improved clinical manifestations in pSS, but their effects on hepatic histopathology in PBC remain to be validated in future clinical studies.

In summary, we describe the common pathways and means of clinical treatment for patients with pSS and PBC by dissecting the serologic and histologic similarities between pSS and PBC. In the future, by delving into the pathogenesis of these two diseases, we may find more effective treatments to provide more effective and personalized therapies for treating patients and improving their quality of life.
